# Pigeon Navigation: Different Routes Lead to Frankfurt

**DOI:** 10.1371/journal.pone.0112439

**Published:** 2014-11-12

**Authors:** Ingo Schiffner, Roswitha Wiltschko

**Affiliations:** 1 FB Biowissenschaften der Goethe-Universität Frankfurt, Siesmayerstraße 70, Frankfurt am Main, Germany; 2 Queensland Brain Institute, University of Queensland, Building #79, St. Lucia, Queensland, Australia; Liverpool John Moores University, United Kingdom

## Abstract

**Background:**

Tracks of pigeons homing to the Frankfurt loft revealed an odd phenomenon: whereas birds returning from the North approach their loft more or less directly in a broad front, pigeons returning from the South choose, from 25 km from home onward, either of two corridors, a direct one and one with a considerable detour to the West. This implies differences in the navigational process.

**Methodology/Principle Findings:**

Pigeons released at sites at the beginning of the westerly corridor and in this corridor behave just like pigeons returning from farther south, deviating to the west before turning towards their loft. Birds released at sites within the straight corridors, in contrast, take more or less straight routes. The analysis of the short-term correlation dimension, a quantity reflecting the complexity of the system and with it, the number of factors involved in the navigational process, reveals that it is significantly larger in pigeons choosing the westerly corridor than in the birds flying straight - 3.03 vs. 2.85. The difference is small, however, suggesting a different interpretation of the same factors, with some birds apparently preferring particular factors over others.

**Conclusions:**

The specific regional distribution of the factors which pigeons use to determine their home course seems to provide ambiguous information in the area 25 km south of the loft, resulting in the two corridors. Pigeons appear to navigate by deriving their routes directly from the locally available navigational factors which they interpret in an individual way. The fractal nature of the correlation dimensions indicates that the navigation process of pigeons is chaotic-deterministic; published tracks of migratory birds suggest that this may apply to avian navigation in general.

## Introduction

When pigeons are released at distant sites, they return to their home loft, but they do not always take the most direct route. In the 1950, when researchers began to observe vanishing bearings, they realized that pigeons mostly take off in directions close to their home direction; at some sites, however, they showed marked deviations from the home course, which turned out to be typical for the respective sites (e.g. [1]–[3]). Keeton [4], analyzing the behavior at such a site, found that his pigeons regularly vanished 60° to 80° clockwise from the home course. He coined the term ‘release site bias’ for these deflections and hypothesized that they were caused by unexpected irregularities in the course of the factors which pigeons use to determine their home course there [3]. After the turn of the century, when GPS recorders had become miniaturized to an extent that pigeon could carry them (e.g. [5], [6]), it became possible to record the entire homing flights with great precision. Analyses of tracks revealed a great variety in homing behavior, with route efficiencies ranging from 0.5 to above 0.9. It became evident that the tracks showed different characteristics in different regions, e.g. route stereotypies and landmark use were reported in England (e.g. [7]–[10]), following linear structures like roads in Italy [11], while neither of this could be observed in Germany [12], [13].

When analyzing tracks of pigeons in the area around our loft at Frankfurt am Main, Germany, we observed an odd phenomenon: whereas birds homing from sites in the North approached their loft more or less directly as one might expect, fanning out a little to the East, birds homing from the South seemed to prefer either of two corridors, a direct one and one with a considerable westerly detour (see e.g. Fig.3 in [14]). Hence we decided to document and analyze this phenomenon in more detail, considering more tracks of pigeons released from the respective directions. In an interdisciplinary approach we use a method derived from dynamic system theory: by time lag embedding, we determined the short-term correlation dimension, a variable indicating the complexity of a system, in order to assess possible changes in the navigational processes en route (see [14]).

### Theoretical Considerations

Time lag embedding is a well-established method in dynamic system theory commonly used to characterize mechanical and mathematical systems [15], [16]. It is beyond the scope of this paper to present a detailed introduction into the theory; for this, we must refer to the many textbooks (see e.g. [17]–[19]). Here we can give only quick overview on the background and how it can be related to pigeon navigation.

### Background

Dynamic systems theory discerns three basic types of systems: deterministic, random and chaotic-deterministic systems. By observation of past states - provided a sufficient amount of observations are available - one can fully predict the behavior of a deterministic system. E. g., observing an ideal pendulum over an entire period provides sufficient knowledge about the pendulum's past states to fully predict any future states of the pendulum - it is a deterministic system. In contrast, when observing a dice, a random system, no amount of observations would grant the observer sufficient information about the behavior of this system – here, knowledge of past states of the system would never yield better predictions about future states than an educated guess.

Chaotic-deterministic systems, although often confused with random systems, are essentially deterministic systems, as suggested by their name. Similar to deterministic systems, observation of past states allows an observer to make predictions about future states of the system. However, due to omnipresent uncertainty combined with a high sensitivity to initial states, which is typical for chaotic-deterministic systems, prediction deteriorates over time until any prediction based on past states is as good as an educated guess, i.e. the system appears to behave randomly. A very common example for chaotic-deterministic systems is the weather: while weather forecasts tend to be reasonably accurate over short periods of time, long term predictions tend to deteriorate quickly.

### Correlation Dimension

The methods we apply to analyze the tracks have been used before to describe biological systems (for review, see [20]). The novelty here is the application to behavioral data – an early attempt can be found in Nehmzow's book on mobile robots [16].

According to Takens' embedding theorem [21], which is an extension of Whitneys' embedding theorem [22], a dynamic system can be fully reconstructed in phase space given a series of observations of the state of the dynamic system, i.e. a time-series. In phase space, every degree of freedom or parameter of the system is represented as an axis in a multi-dimensional space. The number of degrees of freedom then is the minimum number of independent variables necessary to fully describe the system. The advantage of the method compared to other modelling approaches is that it requires no *a priori* knowledge of the underlying system like the number of factors involved and their specific interactions; instead the methods allow us to create a physical model of the process using only the recorded data. The method has been rigorously tested in mathematical systems where the exact number of degrees of freedom is known [19]; there, the number of degrees of freedom is equivalent to the number of terms in the equation. In physical/mechanical systems, it is the number of inputs, e.g. in the case of a robot navigating in a given environment, it is the number of independent sensors necessary to perform this task [16]. - Here we assume that a similar relationship applies to a biological system like a pigeon returning home, with the time series provided by the tracks recorded during the homing flight.

The degrees of freedom of the underlying process, i.e. the number of parameters that vary independently, are reflected by the *correlation dimension*, a value indicating the complexity of the system (for details, see File S1). Applied to pigeon tracks, it provides us with information on the number of independent factors involved, thus, allowing some conclusions on the navigational strategy used [14].

### Navigational Strategies

Adult, experienced pigeons use site-specific information, i.e. local information obtained at the starting point of their return flight, to determine their home direction and the route home [23]. Basically, there are two types of such information a pigeon could use: (1) point-like information tied to a specific location, like e.g. familiar landmarks that characterize a site, and (2) information based on extended geophysical gradients (see [24]–[26] for reviews), like e.g. the geomagnetic field or others. Point-like information can be used in two ways, namely as a direct means of navigation, with the path towards home defined by a sequence of successive landmarks, a strategy called ‘piloting’ [27]; it could also be used as component of a ‘mosaic map’ where each familiar landmark is associated with a compass course from the respective location towards the home loft (see [23], [24], [26], [28]). In both cases, the positions of the respective landmarks have to be learned and remembered in connection with each other. - The use of geophysical gradients is also based on experience: information on the direction and the steepness of the gradients is stored in the navigational ‘map’, which is a directionally oriented mental representation of their spatial distribution, thus allowing pigeons to interpret local values of these gradients with reference to the home value (see [24], [26] for details). Here multiple gradients of different nature could be used simultaneously to increase reliability and precision of that ‘map’ [25], [26]. Unlike point-like information, gradients are not tied to a specific location and can, through extrapolation, provide pigeons with a means to determine the home course even from distant and unfamiliar sites.

The type of information and the strategy used for navigation has direct consequences on the degrees of freedom of the underlying navigational process, which is reflected by the correlation dimension (see [14] for details). If pigeons e.g. use landmarks for piloting, then the underlying process is rather simple: the information provided is merely the path towards the next landmark - this type of information offers only 1 degree of freedom. The mosaic map [23], [24], [28] is also assumed to consist of landmarks, which are static cues and not independent from each other. Each cue within the mosaic map is equivalent to every other. Multiple cues of the same type may increase certainty, but essentially do not provide more information for the navigational process than a single cue alone. Yet the mosaic map also involves at least one additional factor, a compass. Hence navigation based on a mosaic map would result in a higher dimensional process with a correlation dimension of around 2. - If pigeons use environmental gradients as navigational cues, the degrees of freedom of the navigational process depend on the number of gradients involved. Two intersecting gradients, one providing an equivalent to longitudinal and one providing an equivalent to latitudinal information, as well as a compass cue would be the minimum to allow pigeons to find their way home [24], [26]; here, the correlation dimension would be 3, with the involvement of additional factors leading to a further increase.

The correlation dimension can thus indicate the type of navigational strategy used. The analysis of this study is based on the *short-term correlation dimension*, calculated as sliding means over 180 s. It is somewhat lower than the true correlation dimension, but can be used for comparison [14].

## Results

The study is based on 211 tracks from adult, experienced pigeons released at 12 sites, 4 in the North and 8 in the South of the Frankfurt loft (50°08′N, 8°40′E).

### Comparing the tracks from the South and from the North

**Figure 1:**
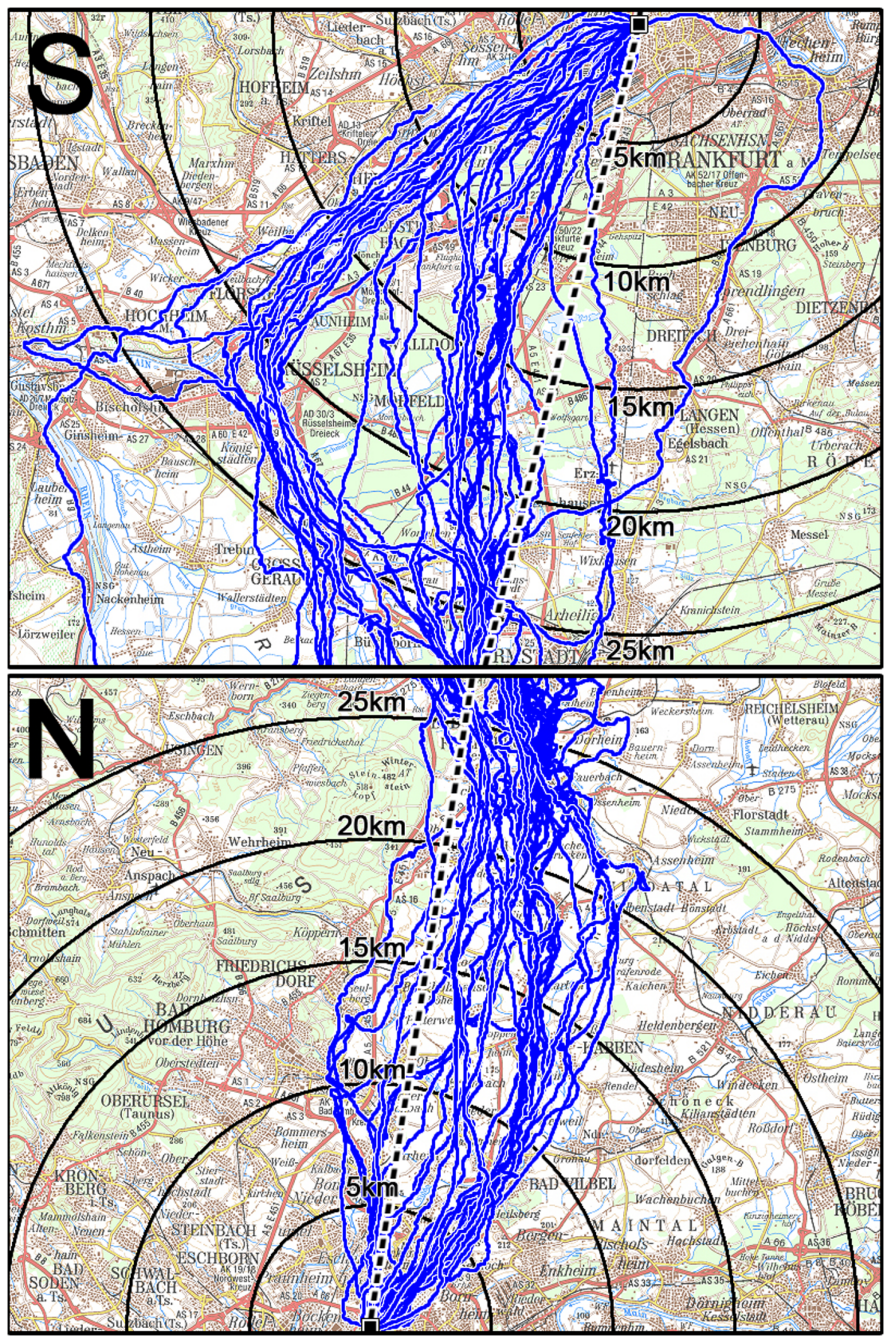
Last 25 km of tracks of pigeons approaching the Frankfurt loft from the South (upper diagram) and from the North (lower diagram). The direct line release point – home is given as a dashed line; tracks are given in blue. The circles indicate the various distances from the loft, which is marked by a black square.

The tracks approaching the loft from the South and from the North within a 25 km radius are documented in Fig. 1. The area the birds crossed when returning from the either direction is rather flat and does not contain any obvious topographical features. Yet a certain difference becomes evident:

The tracks from the South show more scatter compared to those from the North. Slightly before crossing the 25 km radius, they begin to diverge and then concentrate within two distinct corridors, one fairly direct one, lying only slightly west of the beeline, the other swinging out far to the west, with only few intermediate tracks so that the distribution is clearly bimodal. When the pigeons on the westerly corridor reach the area of the valley of the river Main, just before the 20 km radius, they turn northeast, now heading more or less straight towards the loft (Fig. 1, upper diagram). The pigeons homing from the North, in contrast, approached their loft more or less in a broad front. Between 20 to 10 km from home, the tracks cover an area about 10 km wide and then converge towards the loft, with the majority of tracks from about 20 km onward east of the direct line (Fig. 1, lower diagram).

**Figure 2:**
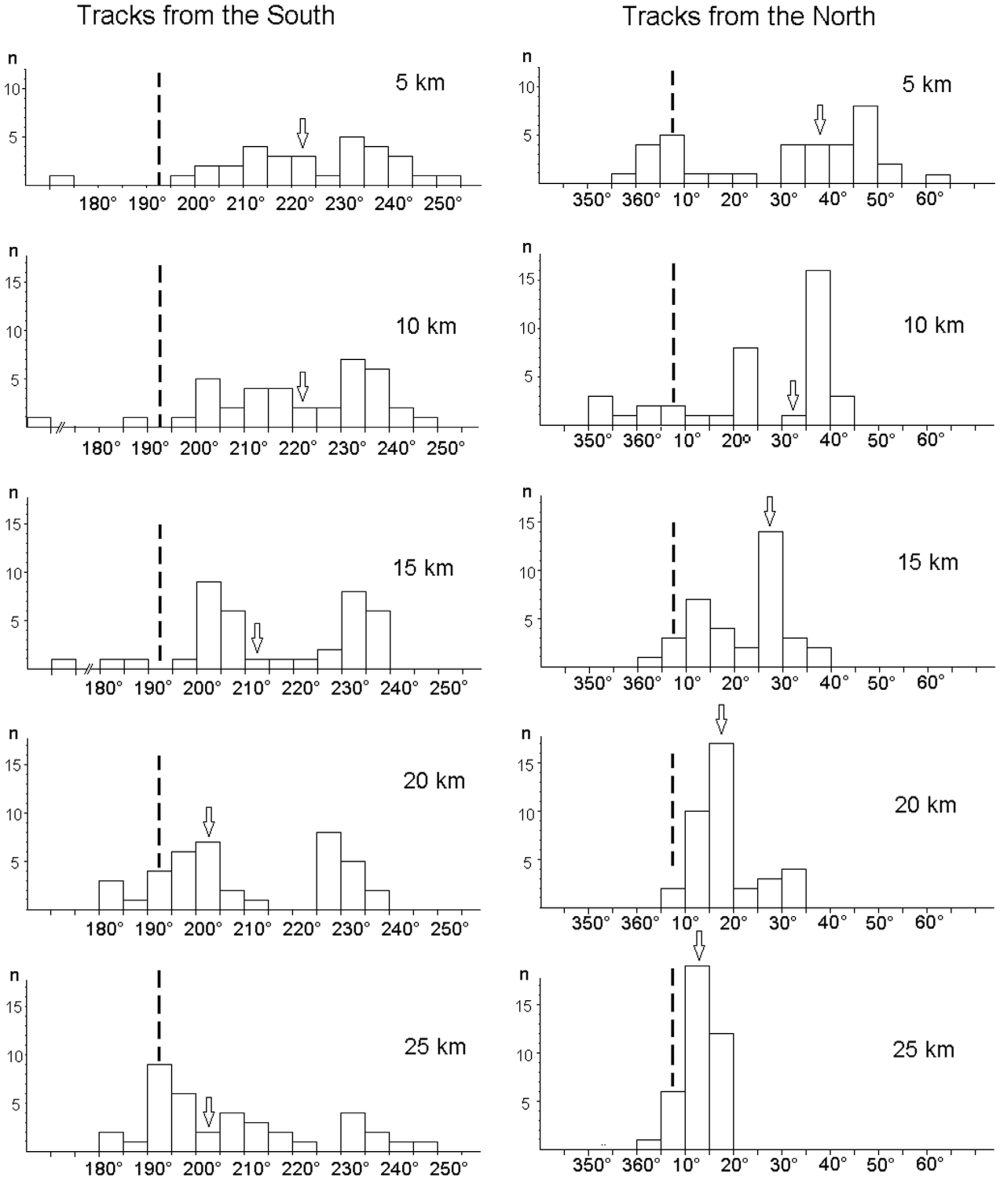
Distribution of bearings at the various distances from the loft; the bearing are given in 5° groups. The dashed line marks the home direction; the open arrow indicates the median of the distribution.

Fig. 2 gives the distribution of the virtual bearings, i.e. the directions of the tracks as seem from the loft when crossing the respective radii, grouped in 5° blocks, at the various distance steps. The tracks from the South show a distinct pattern (see Table 1): the bimodal tendency is visible already at 25 km from the loft; it becomes more pronounced at 20 and 15 km, clearly indicating the two flight corridors, and decreases again from 10 km onward. The tracks from the North, however, are mostly unimodally distributed at the beginning, fanning out as they approach home (due to the fact that the same area covered by the tracks is seen under a larger angle when it is closer to the view point). - A comparison of the distributions of the bearings in the North and in the South with respect to the home direction and with respect to their respective medians gives significant differences at all distances (5 km: p<0.01; other distances: p<0.001, Chi^2^-test; for the test statistics, see Table S1 in File S2), reflecting the different nature of the distributions in the North and the South.

**Table 1:** Deviations from home, short-term correlation dimensions and efficiencies for the various routes.

						Correlation dimension			Median efficiency		
Site	Direction to site	Distance to home	Number of tracks	Vector	Δ home	Median qu1, qu3 (range)			Median qu1, qu3 (range)		
South, all	194°	25 km	38 (30)	209°, 0.954	+15°	2.90	2.71, 3.06	(1.99–3.43)	0.81	0.71. 0.89	(0.62–0.94)
direct corridor			22 (18)	198°, 0,992	+4°	2.85	2.67, 3.00	(1.99–3.18)	0.85	0.72, 0.92	(0.62–0.94)
SW-corridor			16 (12)	230°, 0.996	+36°	3.03 *	2.77, 3.17	(2.62–3.43)	0.75^n.s^	0.69, 0.88	(0.65–0.92)
North, all	9°	25 km	38 (36)	14°, 0.999	+5°	2.80	2.61, 3.14	(2.21–3.64)	0.83	0.76, 0.87	(0.59–0.95)
direct routes			17	13°, 0.998	+4°	3.04	2.76, 3.34	(2.55–3.51)	0.84	0.76, 0.92	(0.68–0.95)
NE routes			21 (19)	33°, 0.996	+24°	2.71 **	2.53, 2.91	(2.21–3.64)	0.82 *	0.73, 0.84	(0.59–0.88)

Number of complete tracks is given in parentheses if different; Vector: for North and South ‘vector’ indicates the mean vector based on the bearings as seen from home when the birds enter the 25 km radius around the loft; for the routes and corridors, it indicates the mean vector of the bearings at the 20 km radius (see text). The median of the correlation dimension, the 1^st^ and 3^rd^ quartiles qu1, qu3 and the range of the correlation dimension are given for all tracks, the respective data for efficiency for all complete tracks. Asterisks at the data from the NE-routes and SW-corridor indicate significant differences between the direct and the deviating routes. - Significance levels by the Mann Whitney U-test:

**, p<0.01;

*, p<0.05; n.s., not significant.

The median short-term correlation dimensions of all tracks from the South and the North, 2.9 and 2.8, respectively (see Table 1) do not differ significantly (u = 0.082; p>0.10, Mann Whitney Test). Yet when considering the birds flying along the corridors separately, we found significant differences between the two corridors in the South, but also between the birds flying directly and those swinging towards east in the North (ANOVA and HSD Tukey test: p<0.001 in both cases; for the overall comparison and details, see Table S2 in File S2). Interestingly, in the South, the birds flying the westerly route have a higher short-term correlation dimension than the ones flying the direct route, whereas in the North, the birds flying directly have a higher short-term correlation dimension (see Table 1).

The efficiencies of the tracks for the last 25 km range from 0.59 to 0.95. The medians are included in Table 1; they did not differ significantly between South and North (u = 0.082, Mann Whitney test). In the South, between the 25 and 20 km radius, however, the birds taking the direct route have a median efficiency of 0.75, flying about 6.7 km to reduce the homeward distance by 5 km, whereas the birds deviating towards west have significantly lower median efficiency of 0.46 (u = 3.37, p<0.001), flying on average 10.9 km. In the North, the efficiencies between 25 and 20 km, with 0.85 for the direct route and 0.63 for the more easterly routes, corresponding to 5.9 and 7.9 km, respectively, also differ significantly (u = 2.86, p<0.01), but here, the difference is considerably smaller.

### Pigeons starting near and on the south-westward corridor and on the direct routes

**Figure 3:**
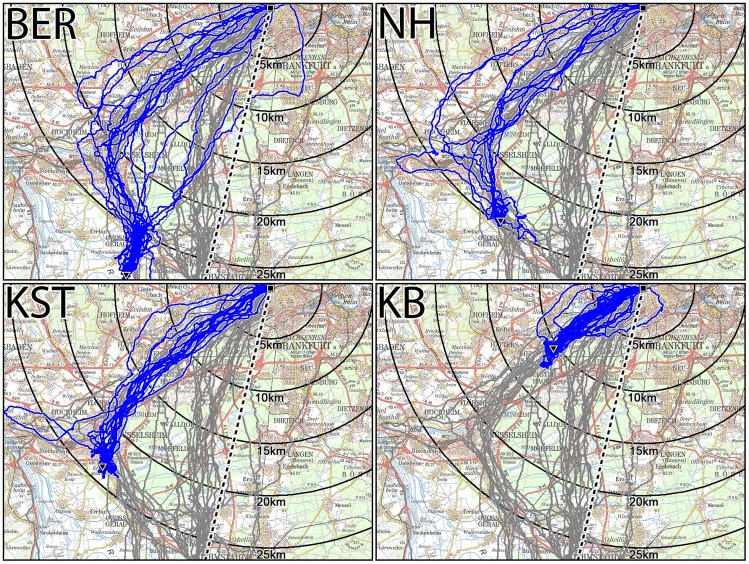
Tracks of birds released at four sites at the beginning (BER), on the westward leg (NH, KST) and on the north-eastward leg of the western corridor (KB). The tracks from the respective sites are given in blue; the tracks of pigeons returning from farther south are indicated in grey for comparison. The release site is marked by a black triangle, the loft by a black square.

Four release sites lie close to the westerly corridor which the pigeons take when homing from the South (Table 2); the tracks from pigeons released at these sites are given in Fig. 3; for the virtual vanishing bearings (i.e. the bearing when the birds crossed the 2.5 km radius around the release point), see Fig. S1 in File S3.

**Table 2:** Virtual bearings, efficiencies and short-term correlation dimensions for the additional sites.

						Correlation dimension			Median efficiency		
Site	Home direction	Distance to home	Number of tracks	Vector	Δ home	Median qu1, qu3 (range)			Median qu1, qu3 (range)		
Berkach	28°	29.5 km	17 (14)	24°, 0.93	- 4°	3.02	2.71, 3.26	(2.58–3.44)	0.64	0.54, 0.71	(0.52–0.89)
Nauheim	33°	25.0 km	10 (9)	344°, 0.82	- −49° *	2.88	2.74, 3.11	(2.24–3.24)	0.73	0.60, 0.81	(0.51–0.88)
Königsstädten	43°	23.5 km	17	7°, 0.95	- 36° *	2.84	2.73, 3.08	(2.17–3.45)	0.84	0.80, 0.88	(0.51–0.91)
Kelsterbach	56°	10.3 km	33 (32)	45°, 0.95	- 11° *	2.85	2.57, 3.03	(2.15–3.47)	0.86	0.78, 0.93	(0.62–0.96)
Gräfenhausen	14°	21.6.km	25 (24)	24°, 0.88	+10°	2.84	2.63, 3.04	(2.14–3.53)	0.84	0.70, 0.92	(0.45–0.97)
Rosbach	194°	19.2 km	33 (32)	199°, 0.94	+5°	2.77	2.49, 3.08	(1.92–3.53)	0.87	0.78, 0.91	(0.41–0.96)

Number of complete tracks is given in parentheses, if different; ‘Vector’ indicates the vector based on the virtual vanishing bearings at 2.5 km from the release point, Δ home, mean deviation from the home direction, with asterisks indicating a significant deviation (p<0.05) (release site bias) as indicated by the confidence interval [54]. The median of the correlation dimension, the quartiles qu1, qu3, and its range are given for all tracks, the respective efficiency data for all complete tracks.

From Berkach, close to where the two southern corridors branch off, the pigeons start homeward oriented, but soon separate into the two corridors. (see Fig. 3, BER). Birds released at the two sites on the westwards leg of the corridor all started out west of the direct route (Fig. 3, NH and KST; see also Fig. S1 in File S3), which leads to a considerable westerly release site bias. Released on the last leg of the western corridor, the pigeons flew more or less straight home (Fig. 3, KB). That is, the pigeons released near and on the westward corridor behaved just like the pigeon that had chosen the westward route when returning from farther south.

**Figure 4:**
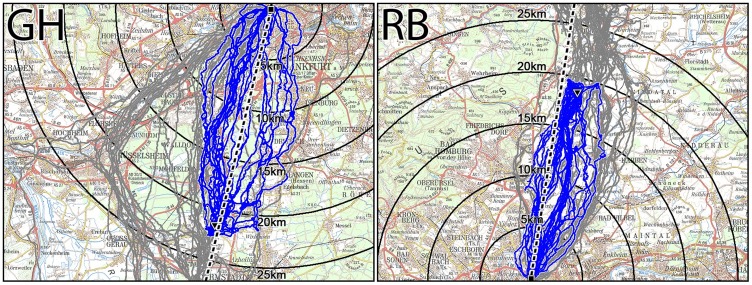
Tracks of pigeons released at sites within the direct corridors: GH in the South and RB in the North. Symbols as in Fig. 3.

The same is true for pigeons released at two sites within the direct corridors (Table 2). The respective tracks are given in Fig. 4: they depart homeward oriented (see Fig. S2 in File S3) and fly more or less directly home.

The short-term correlation dimensions and the efficiencies are included in Table 2. The correlation dimension is highest in the birds starting from Berkach, where the two southern corridors diverge. The efficiencies from the various sites reflect the amount of the detour.

For the original track data on which this analysis is based, see File 4.

## Discussion

Homing from the North, Frankfurt pigeons approach their loft in a broad front with a certain easterly tendency, which is most pronounced 20 to 15 km from the loft. There is no marked gap between the tracks; the transition is smooth and our subdivision appears somewhat arbitrary. When homing from the South, however, birds chose either of two distinct corridors, with the birds that take the westerly corridor flying a marked detour towards west. - How can these peculiar routes be explained?

### The local constellations of navigational factors and their interpretation

Topographical reasons, like e.g. detouring an obstacle (see e.g. [30]) can be largely excluded, because, as already mentioned, the terrain north and south of Frankfurt is rather flat – in the North, there are some very gentle rises, while in the South, where the two corridors are observed, the old flood plain of the river Main is level without any rises. The landscape in the North is dominated by open areas, agriculture and a number of small villages; in the South, there are also villages and fields, but also extended forests. However, it seems rather unlikely that they affect the route choice of pigeons, since both southern corridors pass forests and cross villages and open areas in a similar way. A prominent landmark is visible from any site within the area of our study – the skyline of the city of Frankfurt – but it does not seem to have any effect. The mathematical analysis of the tracks likewise speaks against an involvement of landmarks alone or landmarks together with a compass – the short-term correlation dimensions, indicating a true correlation dimension above 3, are too high to suggest navigation by static cues like landmarks or landscape features alone (see Theoretical Considerations). It indicates navigation by at least three independent factors, for example two gradients and a compass.

When experienced pigeons start their homing flight, they determine their course in a navigational process based on the local values of the available navigational factors. On their way home, they re-determine their home course, – this is evident because pigeons return in spite of large initial deviations caused by release site biases [4] or experimental manipulations like shifting their internal clock (e.g. [31]–[33]). Keeton [4], trying to explain the existence of a pronounced bias and its local constancy, suggested that an unusual distribution of ‘map’ factors at the respective site led the pigeons off the direct route. - Our present study shows that such constellations of ‘map’ factors are effective not only at the beginning of the homing flight, but that they have a direct effect on the routes chosen – they appear to determine the entire homeward route. This means that pigeons are more or less continuously navigating when returning home.

The navigational gradient ‘map’ for long distance orientation is assumed to be a multi-factorial system [25], [26], established by learning processes when the young pigeons begin to fly (see [23], [34]). The nature of the factors included is still not entirely clear; global environmental gradients like e.g. magnetic intensity [35]–[38], Coriolis force [39] and gravity [40], [41] have been suggested, but also odors (e.g. [42]–[46]) and infrasound [47], [48]. It seems also possible that regional cues of only limited range are involved, too, if they prove helpful. In the vicinity of the loft, the gradient ‘map- is supplemented by the mosaic map of landmarks [23], [29]. Pigeons appear to be opportunistic and include in their navigational system all suitable factors available in their home region [25], [26]. How they rate and rank the various factors and which ones they give priority probably depends on their individual early experience during ‘map’ learning (see [34]), so that some individual interpretations are to be expected.

The tracks from the northern sites suggest that the map factors in the area north of Frankfurt are fairly regularly distributed, and even a certain difference in the navigational processes, as indicated by the small difference in correlation dimension, leads only to routes a bit further east. South of Frankfurt, in contrast, the situation is different insofar as in an area in the south-west starting about 25 km from the loft, there seems to be a constellation of local values of ‘map’ factors that is ambiguous and can be interpreted in two different ways. The fairly small difference in short-term correlation dimension suggests that the birds following the two corridors do not use a different number of factors; it rather appears to reflect that the same factors are ranked and rated differently – one interpretation tells pigeons to continue northward, the other that they must change direction and now head north-west. Different individuals decide differently, entering the two distinct corridors. Toward northwest and west of this area, the constellation of ‘map’ factors, appears to lead the birds towards north-west, as indicated by the tracks of the pigeons released at the additional sites along the westerly corridor. Directly in the south of Frankfurt, however, the distribution of ‘map’ factors appears to be more regular, indicating the direct route home, as suggested by the behavior at the site in the direct corridor (GH). The short-term correlation dimensions are high for pigeons starting at Berkach, at the beginning of the westerly corridor; the lower values for birds released along the westerly corridor may indicate a decrease in ambiguous nature of the ‘map’ factors farther north, but they still lead pigeons away from the direct route. - The specific nature of the factors in question must remain open; it is beyond the scope of the present study, and we will refrain from speculations.

### Avian navigation as a chaotic-deterministic process

Analyzing tracks of pigeons by mean of time lag embedding, we found low, but constant Lyapunov exponents [14] and fractal correlation dimensions ([14], [34] and present study), indicating that the navigational process is of a chaotic-deterministic nature. One phenomenon typical for chaotic-deterministic processes is exponential divergence from seemingly similar states and also convergence towards similar states [49]. The tracks presented here also reflect this chaotic-deterministic nature, with pigeons behaving ‘chaotic’ in the sense that they show divergence, i.e. split up into distinctive corridors, and behaving ‘deterministic’ in the sense that they converge and follow the same routes when released along either corridor. Another fine example for divergent routes that converge again at certain points along the way home has been published by Mann and colleagues ([10], see their Fig. 2).

However, this type of behavior does not seem be restricted to homing pigeons. Similar convergence and divergence can also be observed in the routes of migratory birds, especially near stop-over sites, but also elsewhere along the route, so-called intermediary goal areas (see e.g. [50]–[52]). The observation that phenomena typical for chaotic deterministic processes are found in pigeons and migratory birds alike, in flights involving very different distance ranges, suggests that the navigational processes of birds may in general be chaotic-deterministic in nature. - It will be intriguing to analyze tracks of migratory birds with the same methods applied to pigeon tracks here.

### Conclusions

Our data show that the specific distributions of navigational factors encountered on the way home directly affect the navigational decisions and determine the routes chosen. Different interpretations of the same combination of factors may lead to different routes, reflecting individual differences in the rating and ranking of the various factors included in the navigational ‘map. - Time-lag embedding indicates that pigeon homing involves chaotic-deterministic processes; published tracks of migratory birds suggest that this is maybe a general feature of avian navigation.

## Material and Methods

### Ethics Statement

The tracks were recorded under the licences II25 -19c 20/15 – F104/36, V54 19v20/15 – F104/43, V54 19v20/15 – F104/50 and V54 19v20/15 –F104/55 issued by Regierungspräsidium Darmstadt, Land Hessen, Germany.

### Test birds

The analysis is based on tracks of adult, experienced pigeons of our loft at the University of Frankfurt recorded during the summer months of the years 2000, 2001, 2004, 2005, 2006, 2009 and 2010 under sunny weather with little or no wind. The birds – mostly males, because these are stronger and can carry the flight recorder more easily - were between 1 and 10 years old. Their basic experience was similar: during their first year of life, all pigeons had taken part in our standard flock training program up to 40 km in the cardinal compass directions; this training was repeated every spring up to at least 20 to 30 km. The direct routes home from the northern and southern training lines were similar to the direct home lines from the distant sites in the present study. This applies to all our pigeons alike. That is, the area where we analyzed the tracks – within 25 km from the home loft, not far from the training lines - was very familiar to the pigeons. The birds had additional experience of single homing flights from previous tests, the number of varied between individuals and increased with age, but these flights were few compared to the common basic experience of the pigeons and do not influence the behavior within the training range. E.g., at site KS, 17 pigeons between 1 and 10 years old showed excellent agreement in bearings, resulting in a vector of 0.95. On the other hand, of a group of.13 pigeons in their 2^nd^ year of life with homogenous experience returning from the South, seven chose the direct route and six the westerly detour.

### Tracking recorder

The GPS tracking devices used were based on the prototype developed by von Hünerbein et al. [5], later modified and improved by H. Hamann. The recorder was fixed on the pigeon's backs with Velco to the dorsal plate of a harness made from Teflon tape (Bally Ribbon Mills) so that the bird carried it as a backpack (see [12], [13] for details). Although we always made sure that the GPS receiver had contact with a sufficient number of satellites and was properly working before it was wrapped in the plastic coating, technical problems like satellite loss, battery failure, but also pigeons landing occasionally led to data loss and incomplete tracks. In the present study, we included only tracks that were complete or at least had crossed the 10 km radius around the loft. The analysis includes only the first evaluable track of a pigeon from the respective sites (see below).

### Sites used and Analysis of Tracks

We analyzed tracks from three sites in the South with almost the same home direction, and, for comparison, from three sites in the North, with very similar home directions. Table 3 gives the positions of these sites and the number of tracks recorded; the home directions from the South and from the North lie roughly opposite of each other.

**Table 3:** Release sites and number of tracks.

Start site	to site	Distance	Tracks
*In the South*			
Lampertheim	194°	58.9 km	8
Gernsheim	195°	41.8 km	6 (4)
Griesheim	193°	28.8 km	24 (18)
*In the North*			
Reiskirchen	14°	54.5 km	6
Steinfurth	9°	30.7 km	24 (23)
Obermörlen	6°	29.5 km	8 (7)

‘Tracks’ indicates the number of evaluable tracks; in parentheses: number of complete tracks, if different.

Our analysis is based on the routes taken during the last 25 km approaching the home loft. We determined bearings as seen from the loft as the pigeons crossed the 25 km, 20 km, 15 km, 10 km and 5 km radius around the loft. The tracks were then subdivided into subsets defined by the bearing when the bird crossed the 20 km radius: from the South, where the tracks fall into two distinct corridors, the *direct corridor* includes birds that crossed at angles no more than 15° on either side of the home direction, and a *westerly corridor* that includes all birds that crossed farther west, between 20° and 45°. In the North, where the tracks are fairly close together and more or less continuously distributed, we also divided them into two groups to treat them the same way: less than 10° from the home direction for the *direct routes* and between 10° and 25° for the *easterly routes*; - The distributions of these bearings at the various distances in the North and in the South were compared using the Chi^2^ test in relation (1) to the direct course from home, set to 194° from the southern tracks and 9° from the northern ones, and (2) in relation to the respective median bearing.

In order to analyze the phenomenon of the different corridors in more detail, we used tracks from additional sites, five in the South and one in the North; their positions are included in Table 2 in the Result section. Here, we calculated virtual vanishing bearings from the release point when the pigeon crossed the radius 2.5 km from this point (correspond to the traditional vanishing bearings observed with good binoculars) and used the confidence interval [53] to check whether their mean direction was significantly different from home.

### Short-Term Correlation Dimension and Efficiency

Short-term correlation dimensions for the tracks were calculated by means of time lag embedding, a procedure described previously [14]. The calculation is based on the original method first introduced by Grassberger and Procaccia [19], the major difference being that it was calculated as sliding mean over 180 seconds and averaged for each 500 m step from the release site. A summary of the method is given in File S1. Like the correlation dimension, the short-term correlation dimension reflects the number of factors involved in the navigational process (see [14], [16]). For comparing the four subsets of tracks, we used a Two Way ANOVA with a factorial design and repeated measurements, with the Tukey HSD test used for post hoc comparison. Additionally, we took the median for each bird and the second order grand median of these medians to characterize the behavior. For sites beyond 25 km, we only considered the last 25 km; for the additional sites, we excluded the first 2.5 km to eliminate spurious effects from low short-term correlation dimension in the vicinity of the release point (see e.g. [33]).

We also determined the efficiency of the complete tracks; which is defined as direct distance divided by the length of the route taken. Here, too we included the last 25 km and, separately, the routes between the 25 and the 20 km radius, for the additional sites, the overland efficiency from 2.5 km from the release point onward was calculated. Between North and South and between the various routes, these data were compared using the Mann Whitney U-test.

## Supporting Information

File S1 **Calculating the short-term correlation dimension.**
(PDF)

File S2 **Tables giving details on the statistical analysis.** This file includes Table S1 and Table S2. Table S1, Comparing the distribution of bearings in the North and in the South by the Chi^2^-test. Table S2, Testing the short-term correlation dimension of the routes with a Two Way ANOVA.
(PDF)

File S3 **Virtual vanishing bearings, i.e. bearings determined from the tracks at 2.5 km from the release point, at the additional sites.** This file includes Figure S1 and Figure S2. Figure S1, Virtual vanishing at four sites near and on the westerly corridor. Figure S2, Virtual bearings at two sites on the direct corridors.
(PDF)

File S4 **Files containing the original track data and a ‘Read me’ explaining the data format.**
(ZIP)
