# Supplementary material for: Pigeon Navigation: Different Routes Lead to Frankfurt
Source: PLoS One. 2014 Nov 12;9(11):e112439. doi: 10.1371/journal.pone.0112439 (PMC4229201; doi:10.1371/journal.pone.0112439)
Supplement: File S1 — Calculating the short-term correlation dimension. (PDF) [file pone.0112439.s001.pdf]

Supplemental Information 1

Calculating the short-term correlation dimension

In the present study, we focus on the so-called *correlation dimension*, a measurement commonly used in dynamic systems theory (for an introduction into dynamic systems theory, see [S1-S2]). The correlation dimension reflects a system's degrees of freedom [S3], i.e. in our particular case, the number of independent factors involved in the navigational process.

According to Grassberger and Procaccia [S4] the Correlation Integral is defined as:

$$C^m(r) = \frac{1}{M * (M-1)} \sum_{\substack{i,j=1 \\ i \neq j}}^{M} H(r - \|x_i - x_j\|)$$

with x_i and x_j as two points of the reconstructed system $x(t)$, r the distance interval and m the embedding dimension. M is defined as:

$$M = N - (m-1) * \tau$$

With τ the embedding lag ,m the embedding dimension and N the number of data points in the time series.

$H(x)$ denotes the Heavyside step function, defined as:

$$H(x) = \begin{Bmatrix} 1 & x \geq 0 \\ 0 & x < 0 \end{Bmatrix}$$

And finally, ||…|| denotes the Euclidean norm, a measurement of length in Euclidean vector space.

From the correlation integral the correlation dimension D^2 can then be obtained as the slope of ln $[C^m(r)]$ versus $\ln [r]$:

$$D^2 = \lim_{\substack{r \to 0+ \\ m \to \infty}} \frac{\ln[C^m(r)]}{\ln[r]}$$

The calculation, with some minor enhancements implemented to improve data handling for small samples as listed below, was based on the original algorithm proposed by Grassberger and Procaccia [S4] (as shown above); The major difference here is that instead of using all data points, i.e. the entire track, we calculate the correlation dimension over a window of 180 consecutive GPS fixes in a sliding manner. All changes made have been tested extensively with systems where the true fractal dimension is known (see [S5]).

1. In order to ensure *optimal embedding*, we determined the embedding lag for each individual time series. We used the first minimum of the mutual information, a measure of a variables mutual dependence, to determine an appropriate embedding lag [S6] and a fixed range of embedding dimensions - from 3 to 5 - in order to avoid spurious effects from using different embeddings. For the calculation of the *mutual information* we decided to implement a new non-parametric approach in order to circumvent problems with unevenly distributed data. To ascertain that data were always evenly distributed we divided the data into $\log_2$ N (with N being the number of data points in the time series) partitions and assigned a fixed number of elements to each partition. This new approach allowed us to get much better convergence and well defined minima and maxima.

2. In addition we implemented an algorithm for automatic selection of a proper scaling region, thus ensuring that all values would be selected on objective criteria. This was achieved by embedding the time series three times, with successively increasing embedding dimensions. We then choose the scaling region defined by the minimum in standard deviation over all three embeddings.

These changes do not affect the actual calculation of the correlation dimension, but improve data handling and introduce objective criteria for the selection of the scaling region, i.e. the region where the estimate of the correlation dimension is independent of the embedding dimension.

In order to observe changes in the correlation dimension in the course of the homing flight, we determined short-term correlation dimensions for the tracks In contrast to the true correlation dimension based on all data points, they were calculated as sliding means over 180 seconds and averaged for each 500 m step from the release site (see [S7]).. This short-term correlation dimension is lower than the true correlation dimension of the entire track, but can be used for comparisons (see [S4]).

References

S1. Kantz H and Schreiber T (1997). Nonlinear time series analysis. Cambridge University Press, Cambridge.

S2. Kaplan D, Glas, L (1995). Understanding nonlinear dynamics. Springer, New York.

S3. Skinner JE (1994) Low-dimensional chaos in biological systems. Nat Biotechnol 12: 596-600

S4. Grassberger P, Procaccia I (1983) Characterization of strange attractors. Phys Rev Lett 50: 346-349.

S5. Schiffner I (2010) Characterization and Identification of the navigational processes in homing pigeons. PhD dissertation, FB Biowissenschaften, J.W. Goethe-Universität Frankfurt am Main.

S6. Fraser AM, Swinney HL (1986) Independent coordinates for strange attractors from mutual information. Phys Rev A 33: 1134-1140.

S7. Schiffner I, Baumeister J, Wiltschko R (2011) Mathematical analysis of the navigational process in homing pigeons. J Theor Biol 291: 42-46.
