# Supplementary material for: Pigeon Navigation: Different Routes Lead to Frankfurt
Source: PLoS One. 2014 Nov 12;9(11):e112439. doi: 10.1371/journal.pone.0112439 (PMC4229201; doi:10.1371/journal.pone.0112439)
Supplement: File S2 — Tables giving details on the statistical analysis. This file includes Table S1 and Table S2. Table S1, Comparing the distribution of bearings in the North and in the South by the Chi2-test. Table S2, Testing the short-term correlation dimension of the routes with a Two Way ANOVA. (PDF) [file pone.0112439.s002.pdf]

Supplemental Information 2

Tables giving details on the statistical analysis

Table S1. Comparing the distribution of bearings in the North and in the South by the Chi^2-test

	with respect to home			with respect to the median		
Distance	df	Chi^2	sig.	df	Chi^2	sig.
25 km	3	24.08	***	3	19.80	***
20 km	3	22.20	***	3	22.20	***
15 km	3	25.12	***	4	33.52	***
10 km	4	24.20	***	3	21.04	***
5 km	3	12.31	**	3	11.50	**

df, degrees of freedom; under sig., the significance level is indicated: ***, $p < 0.001$; **, p, < 0.01.

Table S2. Testing the short-term correlation dimensions of the routes with a Two Way ANOVA

Table S2a. Results of the ANOVA

	df	Mean of squares	F-value	p	sig.
Distance	49	1.078	5.905	< 0.001	***
Corridors	3	16.675	91.307	< 0.001	***
Interactions	147	0.103	0.566	1.00	n.s.
Residuals	3438	0.183			

Table S2b. Tukey HSD tests comparing the four corridors:

Comparison	difference	(upper,	lower)	p	sig.
N direct - NE	0.269	0.320	0.218	< 0.001	***
N direct – S direct	0.207	0.257	0.156	< 0.001	***
N direct - SW	0.013	0.068	-0.042	0.929	n.s.
NE – S direct	-0.062	-0.014	-0.111	0.005	**
NE - SW	-0.256	-0.203	-0.309	< 0.001	***
S direct - SW	-0.194	-0.141	-0.247	< 0.001	***

'Difference' gives the difference in short-term correlation dimension between the samples tested; '(upper, lower)' indicates the upper and lower limit of the 95% confidence interval; p gives the significance level of the difference, as in Table S1, with n.s., not significant.

The effect of *distance* indicated in Table A2a reflects the increase of the correlation dimension as the pigeons approach home. It was described before [S1] and appears to be a common feature of all tracks. We interpreted it as reflecting an additional use of familiar landmarks in the vicinity of the loft.

Reference

S1. Schiffner I, Baumeister J, Wiltschko R (2011): Mathematical analysis of the navigational process in homing pigeons**.** J Theor Biol 291: 42-46.
