# Supplementary material for: Pigeon Navigation: Different Routes Lead to Frankfurt
Source: PLoS One. 2014 Nov 12;9(11):e112439. doi: 10.1371/journal.pone.0112439 (PMC4229201; doi:10.1371/journal.pone.0112439)
Supplement: File S3 — Virtual vanishing bearings, i.e. bearings determined from the tracks at 2.5 km from the release point, at the additional sites. This file includes Figure S1 and Figure S2. Figure S1, Virtual vanishing at four sites near and on the westerly corridor. Figure S2, Virtual bearings at two sites on the direct corridors. (PDF) [file pone.0112439.s003.pdf]

Supplemental Information 3

Virtual vanishing bearings at the additional sites

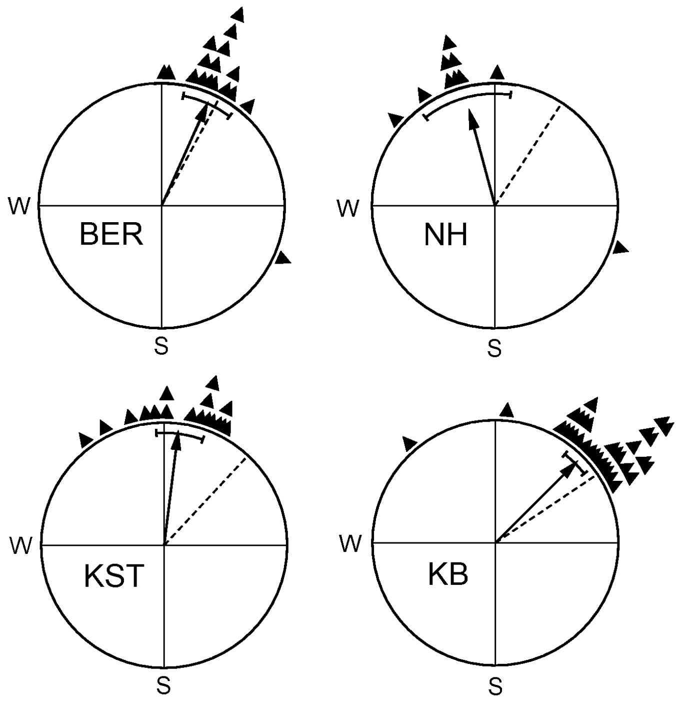

Fig. S1 Virtual vanishing bearings 2.5 km from the release point of pigeons released at four sites near and on the westerly corridor. The respective home directions are marked by a dashed radius. The triangles at the periphery of the circle mark the bearings of individual birds, the arrow represents the mean vector with the length given in relation to the radius of the circle = 1. The small sector at the mean vector indicates the 5% confidence interval.

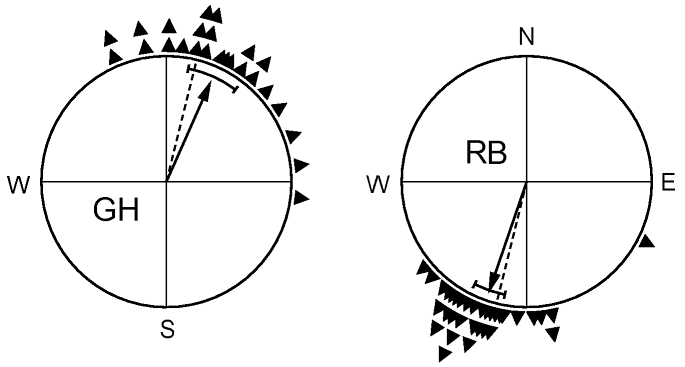

Fig. S2 Virtual bearings 2.5 km from the release point of pigeons released at two sites on the direct corridor. The respective home directions are marked by a dashed radius. The triangles at the periphery of the circle mark the bearings of individual birds, the arrow represents the mean vector with the length given in relation to the radius of the circle = 1. The small sector at the mean vector indicates the 5% confidence interval.
