# Supplementary material for: Pigeon Navigation: Different Routes Lead to Frankfurt
Source: PLoS One. 2014 Nov 12;9(11):e112439. doi: 10.1371/journal.pone.0112439 (PMC4229201; doi:10.1371/journal.pone.0112439)
Supplement: File S4 — Files containing the original track data and a ‘Read me’ explaining the data format. (ZIP) [file pone.0112439.s004.zip › Format of the Track Data.pdf]

Format of the Track Data:

This archive contains GPS data of tracks of pigeons sorted by the two cardinal compass directions relative to the pigeons' home loft. Each folder contains data of pigeons released at a specific site.

Each file follows the same naming conventions:
DATExupBIRD-ID, with the date given in reverse order, i.e. YYMMDD
additional tags at the end of the filename provide information about various failure states
_LATE initial portion of track is missing
_EXC portions of the recording exceed limits for reasonable speed
_GAP track contains gaps in the recording
_INC track is incomplete

Data are stored in ASCII format. Each line contains the data acquired by the GPS at the given time interval. The GPS used records data in propietry format, which differs slightly from the common rmc/nmea standards.

XUP line description:
$GPXUP; GPS day; UTC time; last fix; latitute; longitude; sat count; altitude; speed;

$GPXUP: protocol identifier	String
GPS day: number of days since the 22.08.1999	Integer
UTC time: time of day (Universal Time Convention) without leap seconds	Time
last fix: time in seconds from last fix	Integer
latitude: in decimals (WGS84)	Double
longitude: in decimals (WGS84)	Double
sat count: number of satellites used to acquire the fix	Integer
altitude: altitude in m (if available)	Integer
speed: 2D speed over ground in km/h	Integer
